# A Rare and Intriguing Case of Papillary Thyroid Carcinoma with Tumor Thrombus Extending into the Right Ventricle: Documentation with ^131^ I-NaI-SPECT/CT, MRI, and ^18^ F-FDG-PET/CT

**DOI:** 10.1055/s-0044-1788737

**Published:** 2024-08-05

**Authors:** Parth Baberwal, Ramesh Asopa, Sandip Basu

**Affiliations:** 1 Radiation Medicine Centre, Bhabha Atomic Research Centre, Tata Memorial Hospital Annexe, Jerbai Wadia Road, Parel, Mumbai, India; 2 Homi Bhabha National Institute, Mumbai, India

**Keywords:** tumor thrombus, papillary thyroid carcinoma, radioiodine scan, cardiac MRI, FDG-PET/CT

## Abstract

A unique case of papillary carcinoma of the thyroid with an extensive tumor thrombus extending into the right ventricle is presented. The patient was a known case of solid variant of papillary carcinoma of thyroid, post three cycles of radioiodine therapy, had reported for a diagnostic ^131^ I-NaI scintigraphy as a part of the workup for planning the next ^131^ I therapy. Clinically, the patient was asymptomatic. ^131^ I-NaI scintigraphy showed an arcuate pattern concentration of tracer in the upper mediastinum, which descended up to the lower mediastinum. A ^131^ I-NaI single photon emission computed tomography/computed tomography (SPECT/CT) showed a tracer avid tumor with an extensive tumor thrombus extending from the left brachiocephalic vein to the right ventricle. ^18^ F-fluorodeoxyglucose positron emission tomography/computed tomography ( ^18^ F-FDG-PET/CT) and magnetic resonance imaging (MRI) demonstrated similar findings. The patient was decided to be managed with tyrosine kinase inhibitors as surgical intervention was not deemed possible due to the involvement of major vessels and the high risk of bleeding.

## Introduction

Thyroid gland carcinoma causing tumor thrombus in the great veins of the neck and mediastinum is an uncommon clinical presentation with poor prognosis. 1 To the best of our understanding, only a few cases of papillary carcinoma of thyroid with tumor thrombus extending to heart have been reported in literature. We report a rare case, in a 51-year-old man, of papillary thyroid carcinoma (PTC) with an extensive tumor thrombus extending from the superior vena cava (SVC) to the right ventricle.

## Case Report

**Fig. 1:**
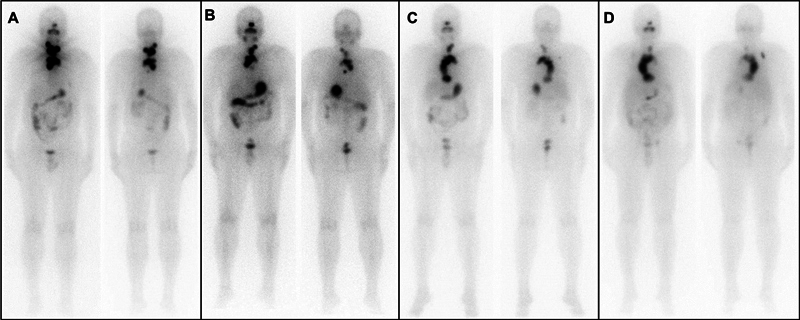
Image-sets (left: anterior acquisition; right: posterior acquisition) of sequential post ^131^ I therapy whole-body planar scintigraphy; A: post 1st therapy; B: post 2 ^nd^ therapy; C: post 3 ^rd^ therapy; D: post 4 ^th^ therapy. Radioiodine refractiveness of the disease and progression of the disease following an arcuate pattern toward the lower mediastinum and reduction of uptake of disease in sequential scans can be noted.

**Fig. 2:**
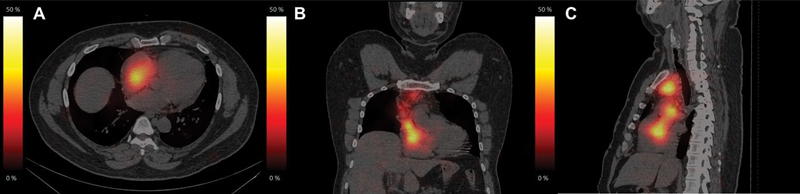
The row of [ ^131^ I]NaI SPECT/CT fused images in axial ( **A** ), coronal ( **B** ) and sagittal views ( **C** ) showing RAI avid tumor thrombus.

**Fig. 3:**
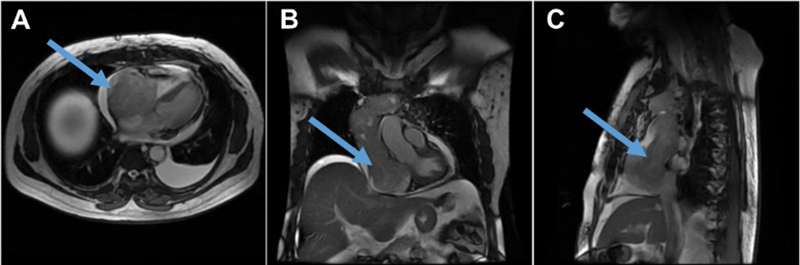
Axial ( **A** ), Coronal ( **B** ) and Sagittal ( **C** ) views of fast imaging employing steady-state acquisition (FIESTA) cardiac MRI sequence images showing intra-ventricular extent of tumor thrombus (marked with blue arrow).

^18^ F-fluorodeoxyglucose positron emission tomography/computed tomography ( ^18^ F-FDG-PET/CT) scan revealed ^18^ F-FDG avid tumor thrombus with an extent to the right ventricle ( Fig. 4 ). Surgical resection was considered but ultimately deemed unfeasible due to the tumor's involvement and adherence to major vessels, posing a high risk of bleeding. The patient complained of facial swelling and upper body edema 1 month post- ^131^ I-NaI therapy. He was initiated on daily oral tyrosine kinase inhibitor (TKI), but succumbed to cardiac failure a few months later.

A 51-year-old gentleman, a known case of solid variant of PTC with nodal metastasis, who underwent three cycles of high dose of adjuvant radioiodine therapy postthyroidectomy, underwent ^131^ I-NaI scintigraphy as part of the follow-up. He was clinically asymptomatic at that time. ^131^ I-NaI scintigraphy showed an archlike uptake in the upper mediastinum, extending caudally to the lower mediastinum ( Fig. 1 ). Stimulated serum thyroglobulin levels were 78,300 ng/mL. ^131^ I-NaI single photon emission computed tomography (SPECT)/CT (non-contrast-enhanced) showed tracer uptake in ill-defined soft-tissue density located in the left brachiocephalic vein extending to the right atrium via the SVC ( Fig. 2 ). The patient underwent one more cycle of adjuvant radioiodine with a dose of 8.17 GBq in view of the aggressive nature of the disease. Echocardiography and cardiac magnetic resonance imaging (MRI) confirmed the presence of an infiltrative mass measuring 16 × 10 × 7 cm encasing the SVC and left brachiocephalic vein infiltrating into the SVC, extending into the right atrium and bulging into the right ventricle. The right ventricular ejection fraction was 61% ( Fig. 3 ).

**Fig. 4:**
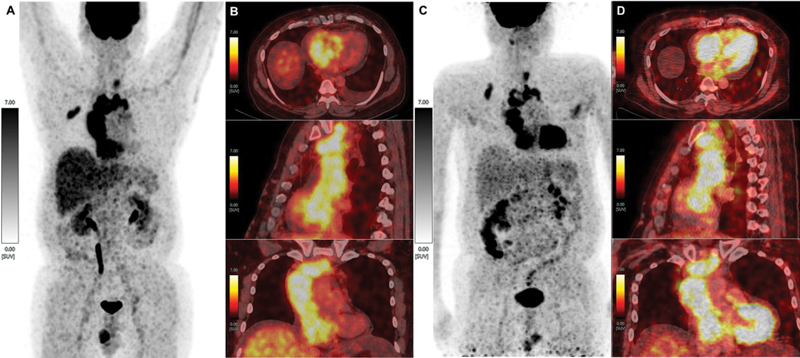
Maximum intensity projection (MIP) of [ ^18^ F]FDG-PET scan which was done prior to 4 ^th^ cycle of [ ^131^ I]NaI administration ( **A** ) and column of images showing axial, coronal and sagittal views (top to bottom) of fused pre-therapy [ ^18^ F]FDG PET/CT, respectively ( **B** ) of [ ^18^ F]FDG avid tumor thrombus. MIP of [ ^18^ F]FDG-PET at the follow-up after RAI administration ( **C** ) and a column of images showing axial, coronal and sagittal views (top to bottom) of post-therapy fused [ ^18^ F]FDG PET/CT, respectively ( **D** ) of [ ^18^ F]FDG-avid tumor thrombus. The serum thyroglobulin at the time of post-RAI [ ^18^ F]FDG PET/CT scan was >350 ng/ml.

## Discussion

There are incidences of the presence of tumor thrombus in the great vessels in cases of aggressive malignancies, which generally have a propensity for hematogenous spread, like Wilm's tumor, renal cell carcinoma, and adrenocortical carcinomas. 2 A review of the literature reported a few cases of tumor thrombus in a major vessel in the setting of thyroid cancer, with the earliest reported in 1879 and the latest in 2023. 3 4 Some of them have been described to have PTC with a tumor thrombus to the right atrium. 5 6 7 8 Gui et al reviewed 47 cases of tumor thrombus in a major vessel in the cases of thyroid carcinoma (in the literature till May 2021), out of which 24 were follicular thyroid carcinoma (FTC), 11 were PTC, 6 were anaplastic thyroid carcinoma (one of them had Hürthle's cell carcinoma coexisting with anaplastic thyroid carcinoma), 3 were Hürthle's cell carcinoma, and 1 was a poorly differentiated thyroid carcinoma. Two of the cases did not have a detailed histopathological report. 9 Ours is the only report of a progressive direct intravascular extension of a tumor thrombus into the right ventricle in a case of PTC with documented progression on ^131^ I-NaI scintigraphy.

In thyroid cancer patients, FTC is known to have a predisposition for vascular invasion and even in that setting, macroscopic tumor thrombi are an uncommon occurrence. In PTC, which has a higher predilection for lymphatic spread, only a few cases of tumor thrombi extending to the right atrium have been reported in the literature, 5 6 7 8 albeit extrathyroidal extension can be noted in 8 to 32% cases of papillary carcinoma of the thyroid. 10

In the cases where the thrombus partially or completely occludes the SVC, the patient may develop symptoms of the SVC syndrome. In the presence of a tumor in a blood vessel, an amenable decision would be to surgically remove the tumor without any residue at baseline. 11 However, in the present case, the patient was put on a trial of TKIs as the intravascular tumor was deemed unresectable.

## Conclusion

In this study, we reported a rare case of radioiodine refractory advanced PTC with subsequent SVC involvement and extension of the tumor thrombus to the right ventricle. The intravascular lesion was unresectable and the patient was started on oral TKI therapy.

## References

[1] Gardner R E Tuttle R M Burman K D Prognostic importance of vascular invasion in papillary thyroid carcinoma Arch Otolaryngol Head Neck Surg 2000 126 03 309 312 10722002 10.1001/archotol.126.3.309

[2] Quencer K B Friedman T Sheth R Oklu R Tumor thrombus: incidence, imaging, prognosis and treatment Cardiovasc Diagn Ther 2017 7 03 S165 S177 29399520 10.21037/cdt.2017.09.16 PMC5778532

[3] Kaufmann C Die Struma Maligna: Primäres Sarkoma und Carcinoma strumae Deutsche Zeitschrift für Chirurgie 1879 11(5–6):401 402

[4] Gill S M Hassan A Bashir H Shafiq W I-131 avid tumor thrombus in a case of poorly differentiated thyroid cancer Mol Imaging Radionucl Ther 2023 32 02 178 180 37337877 10.4274/mirt.galenos.2023.81567 PMC10284192

[5] Yamagami Y Tori M Sakaki M Ohtake S Nakahara M Nakao K Thyroid carcinoma with extensive tumor thrombus in the atrium Gen Thorac Cardiovasc Surg 2008 56 11 555 558 19002756 10.1007/s11748-008-0307-y

[6] Holt W L Extension of malignant tumors of thyroid into great veins and right heart JAMA 1934 102 1921 1924

[7] Hasegawa S Otake Y Bando T Cho H Inui K Wada H Pulmonary dissemination of tumor cells after extended resection of thyroid carcinoma with cardiopulmonary bypass J Thorac Cardiovasc Surg 2002 124 03 635 636 12202885 10.1067/mtc.2002.125060

[8] Sugimoto S Doihara H Ogasawara Y Aoe M Sano S Shimizu N Intraatrial extension of thyroid cancer: a case report Acta Med Okayama 2006 60 02 135 140 16680191 10.18926/AMO/30734

[9] Gui Y Wang J Y Wei X D Middle thyroid vein tumor thrombus in metastatic papillary thyroid microcarcinoma: a case report and review of literature World J Clin Cases 2022 10 10 3213 3221 35647132 10.12998/wjcc.v10.i10.3213 PMC9082703

[10] Mazzaferri E L Jhiang S M Long-term impact of initial surgical and medical therapy on papillary and follicular thyroid cancer Am J Med 1994 97 05 418 428 7977430 10.1016/0002-9343(94)90321-2

[11] Thompson N W Brown J Orringer M Sisson J Nishiyama R Follicular carcinoma of the thyroid with massive angioinvasion: extension of tumor thrombus to the heart Surgery 1978 83 04 451 457 635781

